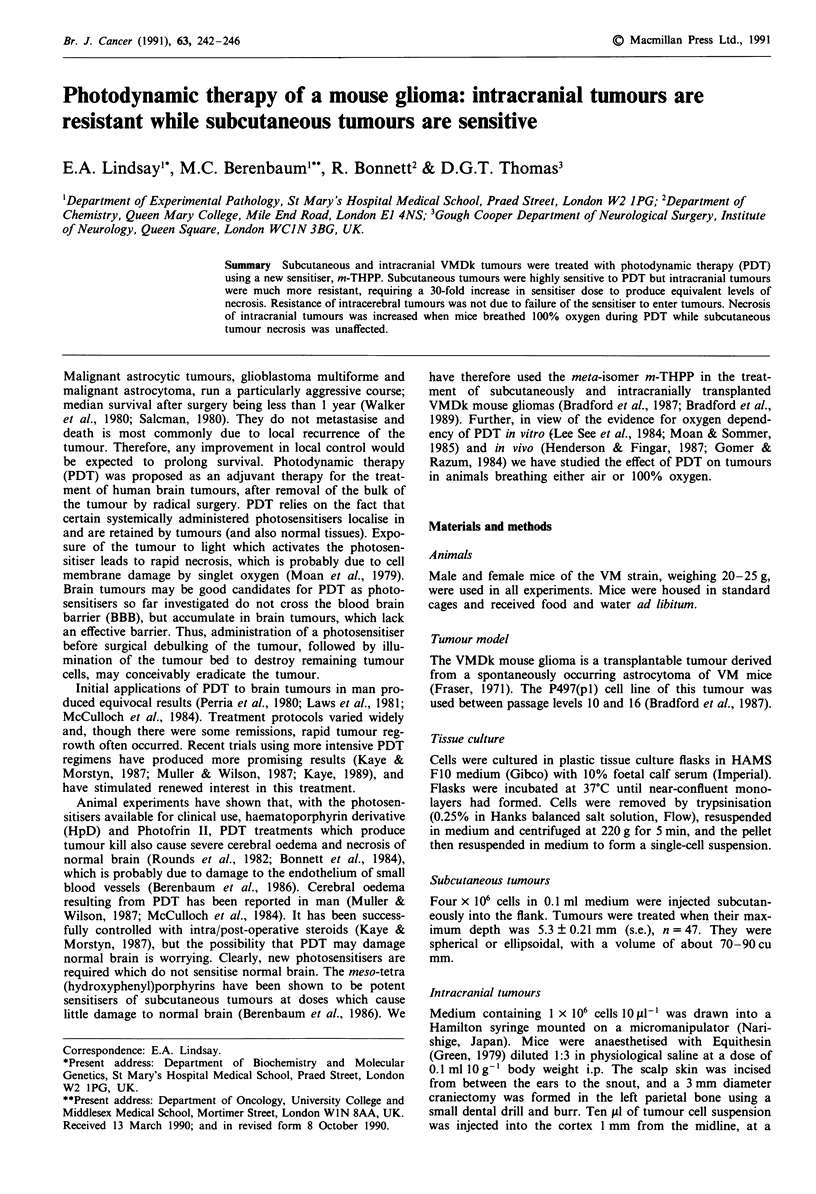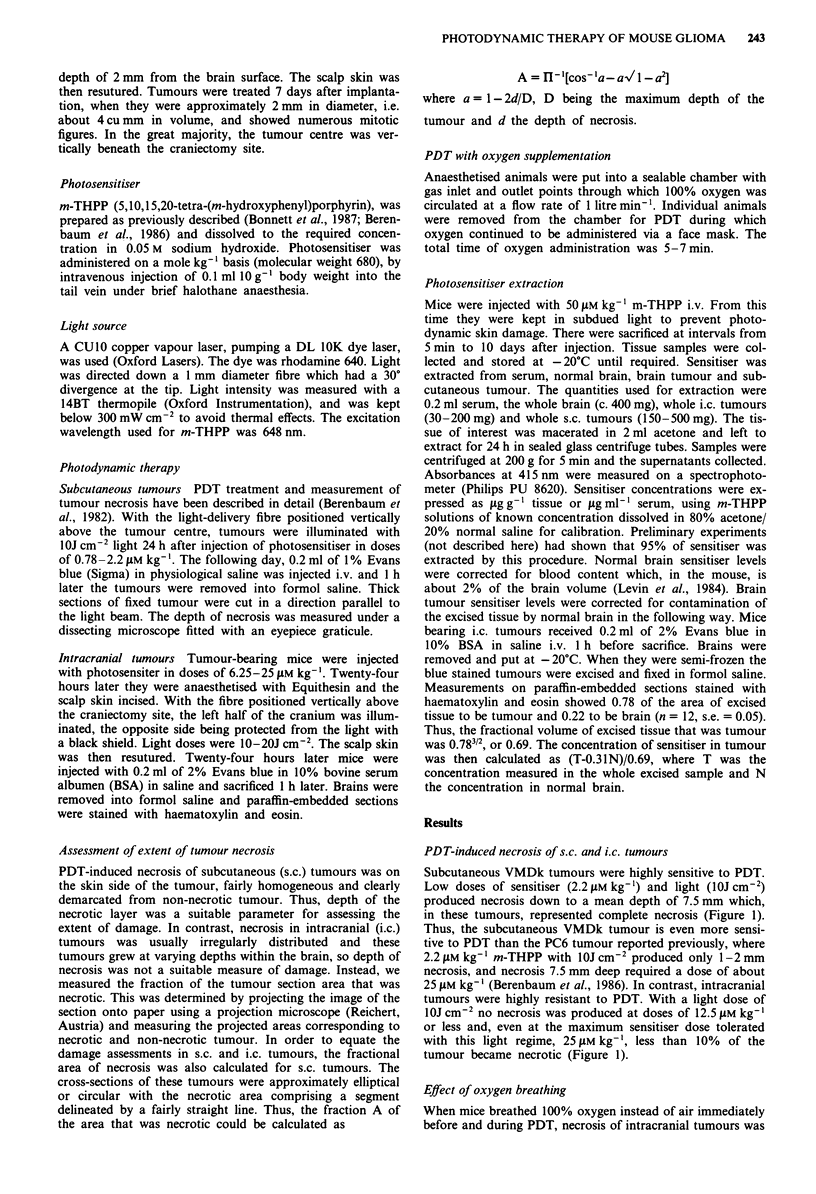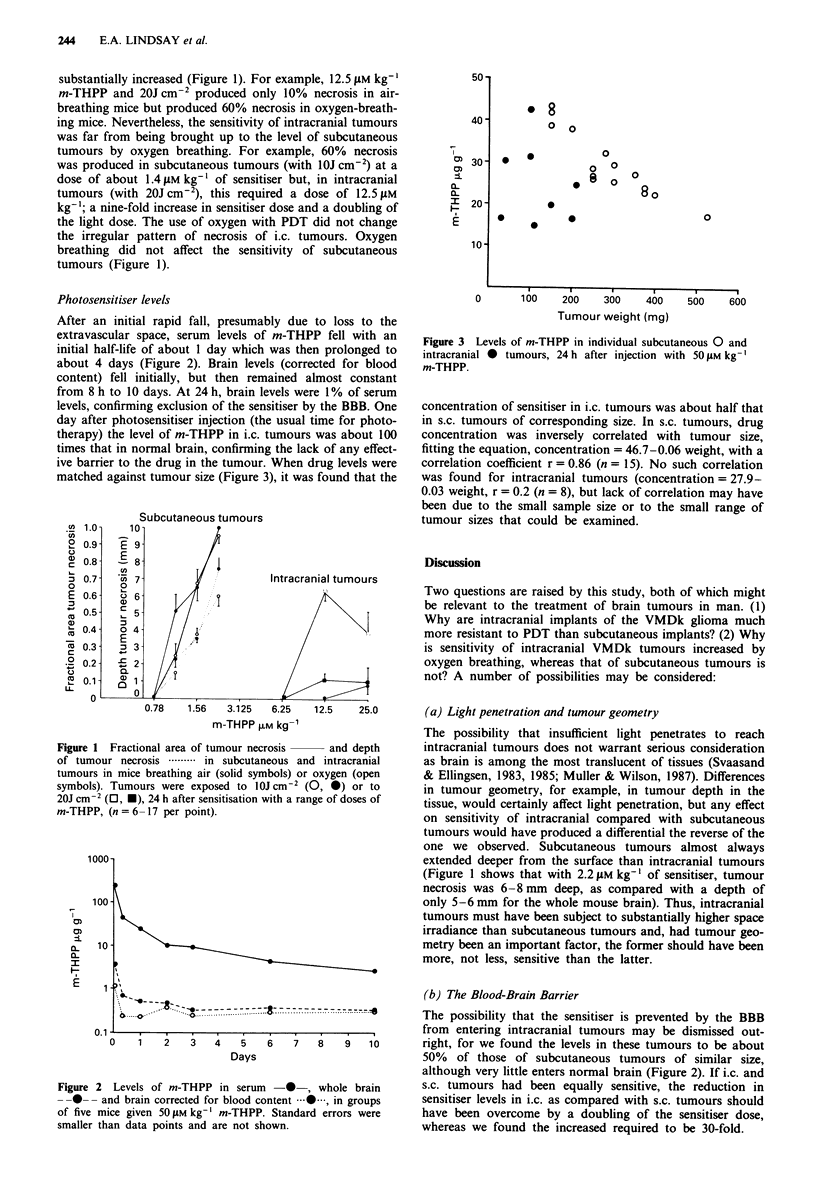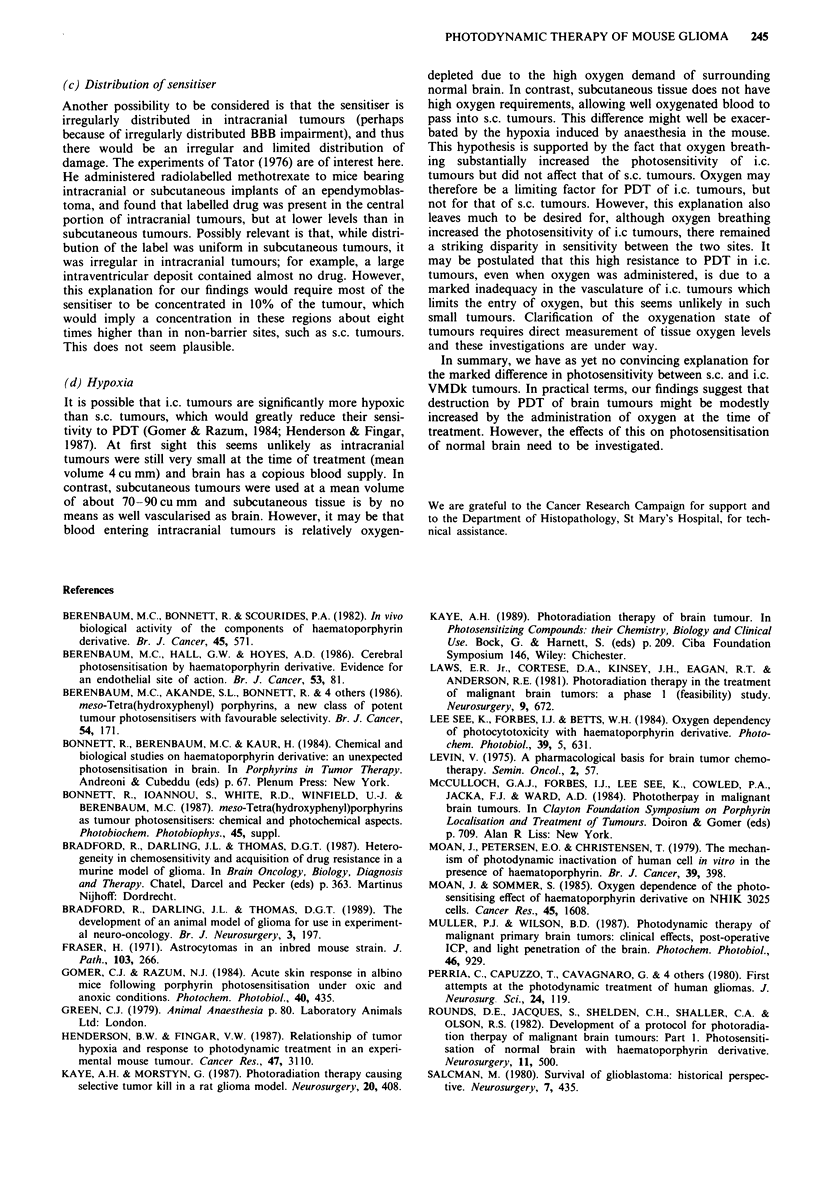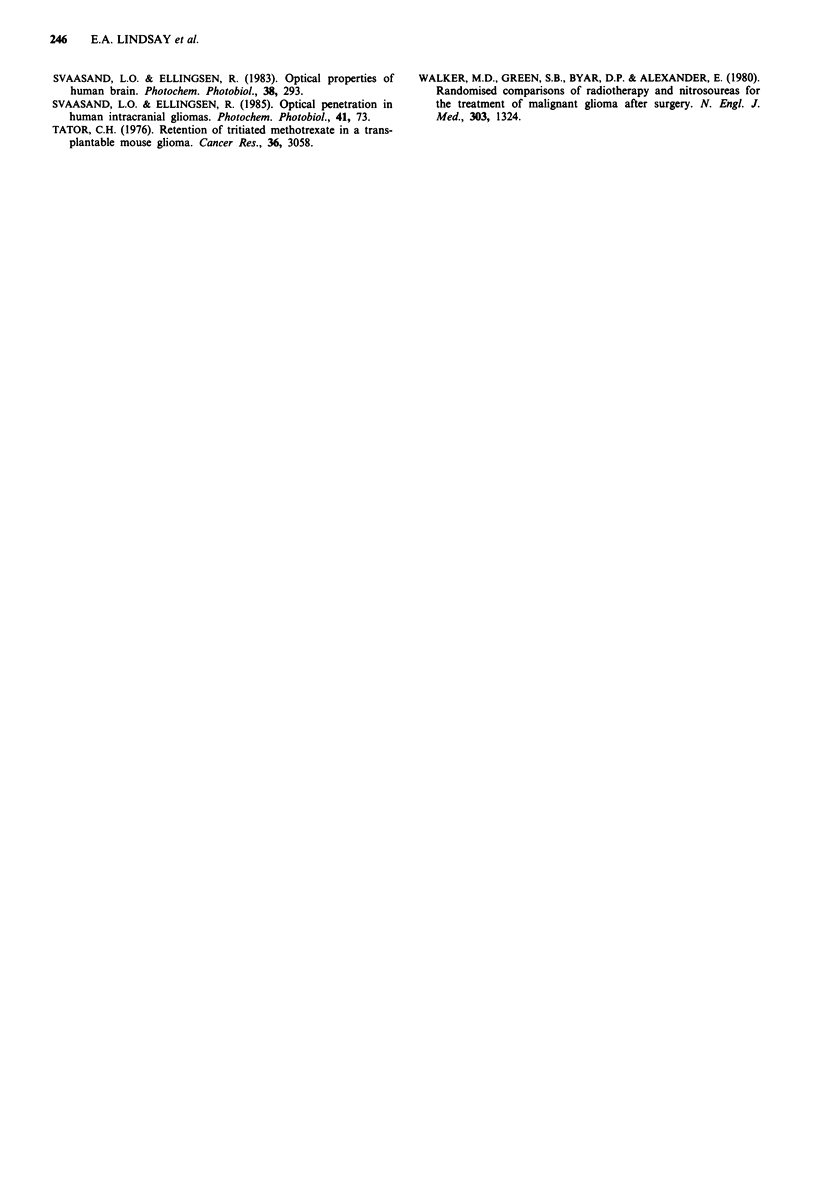# Photodynamic therapy of a mouse glioma: intracranial tumours are resistant while subcutaneous tumours are sensitive.

**DOI:** 10.1038/bjc.1991.57

**Published:** 1991-02

**Authors:** E. A. Lindsay, M. C. Berenbaum, R. Bonnett, D. G. Thomas

**Affiliations:** Department of Experimental Pathology, St Mary's Hospital Medical School, London, UK.

## Abstract

Subcutaneous and intracranial VMDk tumours were treated with photodynamic therapy (PDT) using a new sensitiser, m-THPP. Subcutaneous tumours were highly sensitive to PDT but intracranial tumours were much more resistant, requiring a 30-fold increase in sensitiser dose to produce equivalent levels of necrosis. Resistance of intracerebral tumours was not due to failure of the sensitiser to enter tumours. Necrosis of intracranial tumours was increased when mice breathed 100% oxygen during PDT while subcutaneous tumour necrosis was unaffected.


Br. J. Cancer (1991), 63, 242-246 © Macmillan Press Ltd., 1991

### Photodynamic therapy of a mouse glioma: intracranial tumours are resistant while subcutaneous tumours are sensitive

E.A. Lindsay[1*], M.C. Berenbaum[1**], R. Bonnett[2] & D.G.T. Thomas[3]

[1]*Department of Experimental Pathology, St Mary's Hospital Medical School, Praed Street, London W2 1PG;* [2]*Department of Chemistry, Queen Mary College, Mile End Road, London E1 4NS;* [3]*Gough Cooper Department of Neurological Surgery, Institute of Neurology, Queen Square, London WC1N 3BG, UK.*

**Summary** Subcutaneous and intracranial VMDk tumours were treated with photodynamic therapy (PDT) using a new sensitiser, *m*-THPP. Subcutaneous tumours were highly sensitive to PDT but intracranial tumours were much more resistant, requiring a 30-fold increase in sensitiser dose to produce equivalent levels of necrosis. Resistance of intracerebral tumours was not due to failure of the sensitiser to enter tumours. Necrosis of intracranial tumours was increased when mice breathed 100% oxygen during PDT while subcutaneous tumour necrosis was unaffected.

Malignant astrocytic tumours, glioblastoma multiforme and malignant astrocytoma, run a particularly aggressive course; median survival after surgery being less than 1 year (Walker *et al.*, 1980; Salcman, 1980). They do not metastasise and death is most commonly due to local recurrence of the tumour. Therefore, any improvement in local control would be expected to prolong survival. Photodynamic therapy (PDT) was proposed as an adjuvant therapy for the treatment of human brain tumours, after removal of the bulk of the tumour by radical surgery. PDT relies on the fact that certain systemically administered photosensitisers localise in and are retained by tumours (and also normal tissues). Exposure of the tumour to light which activates the photosensitiser leads to rapid necrosis, which is probably due to cell membrane damage by singlet oxygen (Moan *et al.*, 1979). Brain tumours may be good candidates for PDT as photosensitisers so far investigated do not cross the blood brain barrier (BBB), but accumulate in brain tumours, which lack an effective barrier. Thus, administration of a photosensitiser before surgical debulking of the tumour, followed by illumination of the tumour bed to destroy remaining tumour cells, may conceivably eradicate the tumour.

Initial applications of PDT to brain tumours in man produced equivocal results (Perria *et al.*, 1980; Laws *et al.*, 1981; McCulloch *et al.*, 1984). Treatment protocols varied widely and, though there were some remissions, rapid tumour regrowth often occurred. Recent trials using more intensive PDT regimens have produced more promising results (Kaye & Morstyn, 1987; Muller & Wilson, 1987; Kaye, 1989), and have stimulated renewed interest in this treatment.

Animal experiments have shown that, with the photosensitisers available for clinical use, haematoporphyrin derivative (HpD) and Photofrin II, PDT treatments which produce tumour kill also cause severe cerebral oedema and necrosis of normal brain (Rounds *et al.*, 1982; Bonnett *et al.*, 1984), which is probably due to damage to the endothelium of small blood vessels (Berenbaum *et al.*, 1986). Cerebral oedema resulting from PDT has been reported in man (Muller & Wilson, 1987; McCulloch *et al.*, 1984). It has been successfully controlled with intra/post-operative steroids (Kaye & Morstyn, 1987), but the possibility that PDT may damage normal brain is worrying. Clearly, new photosensitisers are required which do not sensitise normal brain. The *meso*-tetra (hydroxyphenyl)porphyrins have been shown to be potent sensitisers of subcutaneous tumours at doses which cause little damage to normal brain (Berenbaum *et al.*, 1986). We have therefore used the *meta*-isomer *m*-THPP in the treatment of subcutaneously and intracranially transplanted VMDk mouse gliomas (Bradford *et al.*, 1987; Bradford *et al.*, 1989). Further, in view of the evidence for oxygen dependency of PDT *in vitro* (Lee See *et al.*, 1984; Moan & Sommer, 1985) and *in vivo* (Henderson & Fingar, 1987; Gomer & Razum, 1984) we have studied the effect of PDT on tumours in animals breathing either air or 100% oxygen.

### Materials and methods

### *Animals*

Male and female mice of the VM strain, weighing 20-25 g, were used in all experiments. Mice were housed in standard cages and received food and water *ad libitum*.

### *Tumour model*

The VMDk mouse glioma is a transplantable tumour derived from a spontaneously occurring astrocytoma of VM mice (Fraser, 1971). The P497(p1) cell line of this tumour was used between passage levels 10 and 16 (Bradford *et al.*, 1987).

### *Tissue culture*

Cells were cultured in plastic tissue culture flasks in HAMS F10 medium (Gibco) with 10% foetal calf serum (Imperial). Flasks were incubated at 37°C until near-confluent monolayers had formed. Cells were removed by trypsinisation (0.25% in Hanks balanced salt solution, Flow), resuspended in medium and centrifuged at 220 g for 5 min, and the pellet then resuspended in medium to form a single-cell suspension.

### *Subcutaneous tumours*

Four $\times 10^6$ cells in 0.1 ml medium were injected subcutaneously into the flank. Tumours were treated when their maximum depth was $5.3 \pm 0.21$ mm (s.e.), $n = 47$. They were spherical or ellipsoidal, with a volume of about 70-90 cu mm.

### *Intracranial tumours*

Medium containing $1 \times 10^6$ cells $10\,\mu l^{-1}$ was drawn into a Hamilton syringe mounted on a micromanipulator (Narishige, Japan). Mice were anaesthetised with Equithesin (Green, 1979) diluted 1:3 in physiological saline at a dose of $0.1\,ml\,10\,g^{-1}$ body weight i.p. The scalp skin was incised from between the ears to the snout, and a 3 mm diameter craniectomy was formed in the left parietal bone using a small dental drill and burr. Ten μl of tumour cell suspension was injected into the cortex 1 mm from the midline, at a

Correspondence: E.A. Lindsay.
*Present address: Department of Biochemistry and Molecular Genetics, St Mary's Hospital Medical School, Praed Street, London W2 1PG, UK.
**Present address: Department of Oncology, University College and Middlesex Medical School, Mortimer Street, London W1N 8AA, UK.
Received 13 March 1990; and in revised form 8 October 1990.

depth of 2 mm from the brain surface. The scalp skin was then resutured. Tumours were treated 7 days after implantation, when they were approximately 2 mm in diameter, i.e. about 4 cu mm in volume, and showed numerous mitotic figures. In the great majority, the tumour centre was vertically beneath the craniectomy site.

*Photosensitiser*

*m*-THPP (5,10,15,20-tetra-(*m*-hydroxyphenyl)porphyrin), was prepared as previously described (Bonnett *et al.*, 1987; Berenbaum *et al.*, 1986) and dissolved to the required concentration in 0.05 M sodium hydroxide. Photosensitiser was administered on a mole $kg^{-1}$ basis (molecular weight 680), by intravenous injection of 0.1 ml 10 $g^{-1}$ body weight into the tail vein under brief halothane anaesthesia.

*Light source*

A CU10 copper vapour laser, pumping a DL 10K dye laser, was used (Oxford Lasers). The dye was rhodamine 640. Light was directed down a 1 mm diameter fibre which had a 30° divergence at the tip. Light intensity was measured with a 14BT thermopile (Oxford Instrumentation), and was kept below 300 mW $cm^{-2}$ to avoid thermal effects. The excitation wavelength used for *m*-THPP was 648 nm.

*Photodynamic therapy*

*Subcutaneous tumours* PDT treatment and measurement of tumour necrosis have been described in detail (Berenbaum *et al.*, 1982). With the light-delivery fibre positioned vertically above the tumour centre, tumours were illuminated with 10J $cm^{-2}$ light 24 h after injection of photosensitiser in doses of 0.78–2.2 μM $kg^{-1}$. The following day, 0.2 ml of 1% Evans blue (Sigma) in physiological saline was injected i.v. and 1 h later the tumours were removed into formol saline. Thick sections of fixed tumour were cut in a direction parallel to the light beam. The depth of necrosis was measured under a dissecting microscope fitted with an eyepiece graticule.

*Intracranial tumours* Tumour-bearing mice were injected with photosensiter in doses of 6.25–25 μM $kg^{-1}$. Twenty-four hours later they were anaesthetised with Equithesin and the scalp skin incised. With the fibre positioned vertically above the craniectomy site, the left half of the cranium was illuminated, the opposite side being protected from the light with a black shield. Light doses were 10–20J $cm^{-2}$. The scalp skin was then resutured. Twenty-four hours later mice were injected with 0.2 ml of 2% Evans blue in 10% bovine serum albumen (BSA) in saline and sacrificed 1 h later. Brains were removed into formol saline and paraffin-embedded sections were stained with haematoxylin and eosin.

*Assessment of extent of tumour necrosis*

PDT-induced necrosis of subcutaneous (s.c.) tumours was on the skin side of the tumour, fairly homogeneous and clearly demarcated from non-necrotic tumour. Thus, depth of the necrotic layer was a suitable parameter for assessing the extent of damage. In contrast, necrosis in intracranial (i.c.) tumours was usually irregularly distributed and these tumours grew at varying depths within the brain, so depth of necrosis was not a suitable measure of damage. Instead, we measured the fraction of the tumour section area that was necrotic. This was determined by projecting the image of the section onto paper using a projection microscope (Reichert, Austria) and measuring the projected areas corresponding to necrotic and non-necrotic tumour. In order to equate the damage assessments in s.c. and i.c. tumours, the fractional area of necrosis was also calculated for s.c. tumours. The cross-sections of these tumours were approximately elliptical or circular with the necrotic area comprising a segment delineated by a fairly straight line. Thus, the fraction A of the area that was necrotic could be calculated as

$$A = \Pi^{-1}[\cos^{-1}a - a\sqrt{1-a^2}]$$

where $a = 1 - 2d/D$, D being the maximum depth of the tumour and $d$ the depth of necrosis.

*PDT with oxygen supplementation*

Anaesthetised animals were put into a sealable chamber with gas inlet and outlet points through which 100% oxygen was circulated at a flow rate of 1 litre $min^{-1}$. Individual animals were removed from the chamber for PDT during which oxygen continued to be administered via a face mask. The total time of oxygen administration was 5–7 min.

*Photosensitiser extraction*

Mice were injected with 50 μM $kg^{-1}$ m-THPP i.v. From this time they were kept in subdued light to prevent photodynamic skin damage. There were sacrificed at intervals from 5 min to 10 days after injection. Tissue samples were collected and stored at −20°C until required. Sensitiser was extracted from serum, normal brain, brain tumour and subcutaneous tumour. The quantities used for extraction were 0.2 ml serum, the whole brain (c. 400 mg), whole i.c. tumours (30–200 mg) and whole s.c. tumours (150–500 mg). The tissue of interest was macerated in 2 ml acetone and left to extract for 24 h in sealed glass centrifuge tubes. Samples were centrifuged at 200 g for 5 min and the supernatants collected. Absorbances at 415 nm were measured on a spectrophotometer (Philips PU 8620). Sensitiser concentrations were expressed as μg $g^{-1}$ tissue or μg $ml^{-1}$ serum, using *m*-THPP solutions of known concentration dissolved in 80% acetone/20% normal saline for calibration. Preliminary experiments (not described here) had shown that 95% of sensitiser was extracted by this procedure. Normal brain sensitiser levels were corrected for blood content which, in the mouse, is about 2% of the brain volume (Levin *et al.*, 1984). Brain tumour sensitiser levels were corrected for contamination of the excised tissue by normal brain in the following way. Mice bearing i.c. tumours received 0.2 ml of 2% Evans blue in 10% BSA in saline i.v. 1 h before sacrifice. Brains were removed and put at −20°C. When they were semi-frozen the blue stained tumours were excised and fixed in formol saline. Measurements on paraffin-embedded sections stained with haematoxylin and eosin showed 0.78 of the area of excised tissue to be tumour and 0.22 to be brain ($n$ = 12, s.e. = 0.05). Thus, the fractional volume of excised tissue that was tumour was $0.78^{3/2}$, or 0.69. The concentration of sensitiser in tumour was then calculated as (T-0.31N)/0.69, where T was the concentration measured in the whole excised sample and N the concentration in normal brain.

### Results

*PDT-induced necrosis of s.c. and i.c. tumours*

Subcutaneous VMDk tumours were highly sensitive to PDT. Low doses of sensitiser (2.2 μM $kg^{-1}$) and light (10J $cm^{-2}$) produced necrosis down to a mean depth of 7.5 mm which, in these tumours, represented complete necrosis (Figure 1). Thus, the subcutaneous VMDk tumour is even more sensitive to PDT than the PC6 tumour reported previously, where 2.2 μM $kg^{-1}$ *m*-THPP with 10J $cm^{-2}$ produced only 1–2 mm necrosis, and necrosis 7.5 mm deep required a dose of about 25 μM $kg^{-1}$ (Berenbaum *et al.*, 1986). In contrast, intracranial tumours were highly resistant to PDT. With a light dose of 10J $cm^{-2}$ no necrosis was produced at doses of 12.5 μM $kg^{-1}$ or less and, even at the maximum sensitiser dose tolerated with this light regime, 25 μM $kg^{-1}$, less than 10% of the tumour became necrotic (Figure 1).

*Effect of oxygen breathing*

When mice breathed 100% oxygen instead of air immediately before and during PDT, necrosis of intracranial tumours was substantially increased (Figure 1). For example, 12.5 μM $kg^{-1}$ *m*-THPP and 20J $cm^{-2}$ produced only 10% necrosis in air-breathing mice but produced 60% necrosis in oxygen-breathing mice. Nevertheless, the sensitivity of intracranial tumours was far from being brought up to the level of subcutaneous tumours by oxygen breathing. For example, 60% necrosis was produced in subcutaneous tumours (with 10J $cm^{-2}$) at a dose of about 1.4 μM $kg^{-1}$ of sensitiser but, in intracranial tumours (with 20J $cm^{-2}$), this required a dose of 12.5 μM $kg^{-1}$; a nine-fold increase in sensitiser dose and a doubling of the light dose. The use of oxygen with PDT did not change the irregular pattern of necrosis of i.c. tumours. Oxygen breathing did not affect the sensitivity of subcutaneous tumours (Figure 1).

*Photosensitiser levels*

After an initial rapid fall, presumably due to loss to the extravascular space, serum levels of *m*-THPP fell with an initial half-life of about 1 day which was then prolonged to about 4 days (Figure 2). Brain levels (corrected for blood content) fell initially, but then remained almost constant from 8 h to 10 days. At 24 h, brain levels were 1% of serum levels, confirming exclusion of the sensitiser by the BBB. One day after photosensitiser injection (the usual time for phototherapy) the level of *m*-THPP in i.c. tumours was about 100 times that in normal brain, confirming the lack of any effective barrier to the drug in the tumour. When drug levels were matched against tumour size (Figure 3), it was found that the concentration of sensitiser in i.c. tumours was about half that in s.c. tumours of corresponding size. In s.c. tumours, drug concentration was inversely correlated with tumour size, fitting the equation, concentration = 46.7–0.06 weight, with a correlation coefficient r = 0.86 ($n = 15$). No such correlation was found for intracranial tumours (concentration = 27.9–0.03 weight, r = 0.2 ($n = 8$), but lack of correlation may have been due to the small sample size or to the small range of tumour sizes that could be examined.

**Figure 1** Fractional area of tumour necrosis ——— and depth of tumour necrosis ········ in subcutaneous and intracranial tumours in mice breathing air (solid symbols) or oxygen (open symbols). Tumours were exposed to 10J $cm^{-2}$ (O, ●) or to 20J $cm^{-2}$ (□, ■), 24 h after sensitisation with a range of doses of *m*-THPP, ($n = 6–17$ per point).

**Figure 2** Levels of *m*-THPP in serum —●—, whole brain --●-- and brain corrected for blood content ···●···, in groups of five mice given 50 μM $kg^{-1}$ *m*-THPP. Standard errors were smaller than data points and are not shown.

**Figure 3** Levels of *m*-THPP in individual subcutaneous O and intracranial ● tumours, 24 h after injection with 50 μM $kg^{-1}$ *m*-THPP.

### Discussion

Two questions are raised by this study, both of which might be relevant to the treatment of brain tumours in man. (1) Why are intracranial implants of the VMDk glioma much more resistant to PDT than subcutaneous implants? (2) Why is sensitivity of intracranial VMDk tumours increased by oxygen breathing, whereas that of subcutaneous tumours is not? A number of possibilities may be considered:

*(a) Light penetration and tumour geometry*

The possibility that insufficient light penetrates to reach intracranial tumours does not warrant serious consideration as brain is among the most translucent of tissues (Svaasand & Ellingsen, 1983, 1985; Muller & Wilson, 1987). Differences in tumour geometry, for example, in tumour depth in the tissue, would certainly affect light penetration, but any effect on sensitivity of intracranial compared with subcutaneous tumours would have produced a differential the reverse of the one we observed. Subcutaneous tumours almost always extended deeper from the surface than intracranial tumours (Figure 1 shows that with 2.2 μM $kg^{-1}$ of sensitiser, tumour necrosis was 6–8 mm deep, as compared with a depth of only 5–6 mm for the whole mouse brain). Thus, intracranial tumours must have been subject to substantially higher space irradiance than subcutaneous tumours and, had tumour geometry been an important factor, the former should have been more, not less, sensitive than the latter.

*(b) The Blood-Brain Barrier*

The possibility that the sensitiser is prevented by the BBB from entering intracranial tumours may be dismissed outright, for we found the levels in these tumours to be about 50% of those of subcutaneous tumours of similar size, although very little enters normal brain (Figure 2). If i.c. and s.c. tumours had been equally sensitive, the reduction in sensitiser levels in i.c. as compared with s.c. tumours should have been overcome by a doubling of the sensitiser dose, whereas we found the increased required to be 30-fold.

*(c) Distribution of sensitiser*

Another possibility to be considered is that the sensitiser is irregularly distributed in intracranial tumours (perhaps because of irregularly distributed BBB impairment), and thus there would be an irregular and limited distribution of damage. The experiments of Tator (1976) are of interest here. He administered radiolabelled methotrexate to mice bearing intracranial or subcutaneous implants of an ependymoblastoma, and found that labelled drug was present in the central portion of intracranial tumours, but at lower levels than in subcutaneous tumours. Possibly relevant is that, while distribution of the label was uniform in subcutaneous tumours, it was irregular in intracranial tumours; for example, a large intraventricular deposit contained almost no drug. However, this explanation for our findings would require most of the sensitiser to be concentrated in 10% of the tumour, which would imply a concentration in these regions about eight times higher than in non-barrier sites, such as s.c. tumours. This does not seem plausible.

*(d) Hypoxia*

It is possible that i.c. tumours are significantly more hypoxic than s.c. tumours, which would greatly reduce their sensitivity to PDT (Gomer & Razum, 1984; Henderson & Fingar, 1987). At first sight this seems unlikely as intracranial tumours were still very small at the time of treatment (mean volume 4 cu mm) and brain has a copious blood supply. In contrast, subcutaneous tumours were used at a mean volume of about 70–90 cu mm and subcutaneous tissue is by no means as well vascularised as brain. However, it may be that blood entering intracranial tumours is relatively oxygen-depleted due to the high oxygen demand of surrounding normal brain. In contrast, subcutaneous tissue does not have high oxygen requirements, allowing well oxygenated blood to pass into s.c. tumours. This difference might well be exacerbated by the hypoxia induced by anaesthesia in the mouse. This hypothesis is supported by the fact that oxygen breathing substantially increased the photosensitivity of i.c. tumours but did not affect that of s.c. tumours. Oxygen may therefore be a limiting factor for PDT of i.c. tumours, but not for that of s.c. tumours. However, this explanation also leaves much to be desired for, although oxygen breathing increased the photosensitivity of i.c tumours, there remained a striking disparity in sensitivity between the two sites. It may be postulated that this high resistance to PDT in i.c. tumours, even when oxygen was administered, is due to a marked inadequacy in the vasculature of i.c. tumours which limits the entry of oxygen, but this seems unlikely in such small tumours. Clarification of the oxygenation state of tumours requires direct measurement of tissue oxygen levels and these investigations are under way.

In summary, we have as yet no convincing explanation for the marked difference in photosensitivity between s.c. and i.c. VMDk tumours. In practical terms, our findings suggest that destruction by PDT of brain tumours might be modestly increased by the administration of oxygen at the time of treatment. However, the effects of this on photosensitisation of normal brain need to be investigated.

We are grateful to the Cancer Research Campaign for support and to the Department of Histopathology, St Mary's Hospital, for technical assistance.

**References**

BERENBAUM, M.C., BONNETT, R. & SCOURIDES, P.A. (1982). *In vivo* biological activity of the components of haematoporphyrin derivative. *Br. J. Cancer*, **45**, 571.

BERENBAUM, M.C., HALL, G.W. & HOYES, A.D. (1986). Cerebral photosensitisation by haematoporphyrin derivative. Evidence for an endothelial site of action. *Br. J. Cancer*, **53**, 81.

BERENBAUM, M.C., AKANDE, S.L., BONNETT, R. & 4 others (1986). *meso*-Tetra(hydroxyphenyl) porphyrins, a new class of potent tumour photosensitisers with favourable selectivity. *Br. J. Cancer*, **54**, 171.

BONNETT, R., BERENBAUM, M.C. & KAUR, H. (1984). Chemical and biological studies on haematoporphyrin derivative: an unexpected photosensitisation in brain. In *Porphyrins in Tumor Therapy*. Andreoni & Cubeddu (eds) p. 67. Plenum Press: New York.

BONNETT, R., IOANNOU, S., WHITE, R.D., WINFIELD, U.-J. & BERENBAUM, M.C. (1987). *meso*-Tetra(hydroxyphenyl)porphyrins as tumour photosensitisers: chemical and photochemical aspects. *Photobiochem. Photobiophys.*, **45**, suppl.

BRADFORD, R., DARLING, J.L. & THOMAS, D.G.T. (1987). Heterogeneity in chemosensitivity and acquisition of drug resistance in a murine model of glioma. In *Brain Oncology, Biology, Diagnosis and Therapy*. Chatel, Darcel and Pecker (eds) p. 363. Martinus Nijhoff: Dordrecht.

BRADFORD, R., DARLING, J.L. & THOMAS, D.G.T. (1989). The development of an animal model of glioma for use in experimental neuro-oncology. *Br. J. Neurosurgery*, **3**, 197.

FRASER, H. (1971). Astrocytomas in an inbred mouse strain. *J. Path.*, **103**, 266.

GOMER, C.J. & RAZUM, N.J. (1984). Acute skin response in albino mice following porphyrin photosensitisation under oxic and anoxic conditions. *Photochem. Photobiol.*, **40**, 435.

GREEN, C.J. (1979). *Animal Anaesthesia* p. 80. Laboratory Animals Ltd: London.

HENDERSON, B.W. & FINGAR, V.W. (1987). Relationship of tumor hypoxia and response to photodynamic treatment in an experimental mouse tumour. *Cancer Res.*, **47**, 3110.

KAYE, A.H. & MORSTYN, G. (1987). Photoradiation therapy causing selective tumor kill in a rat glioma model. *Neurosurgery*, **20**, 408.

KAYE, A.H. (1989). Photoradiation therapy of brain tumour. In *Photosensitizing Compounds: their Chemistry, Biology and Clinical Use*. Bock, G. & Harnett, S. (eds) p. 209. Ciba Foundation Symposium 146, Wiley: Chichester.

LAWS, E.R. Jr., CORTESE, D.A., KINSEY, J.H., EAGAN, R.T. & ANDERSON, R.E. (1981). Photoradiation therapy in the treatment of malignant brain tumors: a phase 1 (feasibility) study. *Neurosurgery*, **9**, 672.

LEE SEE, K., FORBES, I.J. & BETTS, W.H. (1984). Oxygen dependency of photocytotoxicity with haematoporphyrin derivative. *Photochem. Photobiol.*, **39**, 5, 631.

LEVIN, V. (1975). A pharmacological basis for brain tumor chemotherapy. *Semin. Oncol.*, **2**, 57.

McCULLOCH, G.A.J., FORBES, I.J., LEE SEE, K., COWLED, P.A., JACKA, F.J. & WARD, A.D. (1984). Phototherpay in malignant brain tumours. In *Clayton Foundation Symposium on Porphyrin Localisation and Treatment of Tumours*. Doiron & Gomer (eds) p. 709. Alan R Liss: New York.

MOAN, J., PETERSEN, E.O. & CHRISTENSEN, T. (1979). The mechanism of photodynamic inactivation of human cell *in vitro* in the presence of haematoporphyrin. *Br. J. Cancer*, **39**, 398.

MOAN, J. & SOMMER, S. (1985). Oxygen dependence of the photosensitising effect of haematoporphyrin derivative on NHIK 3025 cells. *Cancer Res.*, **45**, 1608.

MULLER, P.J. & WILSON, B.D. (1987). Photodynamic therapy of malignant primary brain tumors: clinical effects, post-operative ICP, and light penetration of the brain. *Photochem. Photobiol.*, **46**, 929.

PERRIA, C., CAPUZZO, T., CAVAGNARO, G. & 4 others (1980). First attempts at the photodynamic treatment of human gliomas. *J. Neurosurg. Sci.*, **24**, 119.

ROUNDS, D.E., JACQUES, S., SHELDEN, C.H., SHALLER, C.A. & OLSON, R.S. (1982). Development of a protocol for photoradiation therpay of malignant brain tumours: Part 1. Photosensitisation of normal brain with haematoporphyrin derivative. *Neurosurgery*, **11**, 500.

SALCMAN, M. (1980). Survival of glioblastoma: historical perspective. *Neurosurgery*, **7**, 435.

SVAASAND, L.O. & ELLINGSEN, R. (1983). Optical properties of human brain. *Photochem. Photobiol.*, **38**, 293.

SVAASAND, L.O. & ELLINGSEN, R. (1985). Optical penetration in human intracranial gliomas. *Photochem. Photobiol.*, **41**, 73.

TATOR, C.H. (1976). Retention of tritiated methotrexate in a transplantable mouse glioma. *Cancer Res.*, **36**, 3058.

WALKER, M.D., GREEN, S.B., BYAR, D.P. & ALEXANDER, E. (1980). Randomised comparisons of radiotherapy and nitrosoureas for the treatment of malignant glioma after surgery. *N. Engl. J. Med.*, **303**, 1324.